# Water-Transmitted Fungi Are Involved in Degradation of Concrete Drinking Water Storage Tanks

**DOI:** 10.3390/microorganisms9010160

**Published:** 2021-01-12

**Authors:** Monika Novak Babič, Nina Gunde-Cimerman

**Affiliations:** Department of Biology, Biotechnical Faculty, University of Ljubljana, SI-1000 Ljubljana, Slovenia; nina.gunde-cimerman@bf.uni-lj.si

**Keywords:** degradation, chlorination, drinking water, environmental factors, fungal contaminants, growth of fungi, materials in contact with drinking water, water storage tanks

## Abstract

Global warming, globalization, industrialization, and the rapidly growing population at present increasingly affect the production of safe drinking water. In combination with sustainable bio-based or recycled materials, used for water distribution systems, these factors promote emerging pathogens, including fungi. They can proliferate in oligotrophic water systems, affect the disinfection process, degrade building materials, and cause diseases in humans. In this study, we explored fungal-based degradation of modern concrete water storage tanks and the presence of fungi in chlorinated drinking water at the entrance and exit of the tanks. The degradation potential of isolated 52 fungal strains and their growth at different oligotrophic conditions was tested in vitro. Forty percent of strains grew at extremely oligotrophic conditions, and 50% classified as aerophilic. Two-thirds of tested strains produced acids, with *Penicillium* strains as the best producers. Only 29.7% of the strains were able to grow at 37 °C, and none of them was isolated from drinking water at consumers’ taps. Although not yet part of the guidelines for building materials in contact with drinking water, fungi should be taken into consideration in case of visible degradation. Their number of consumers’ endpoints should be checked to exclude possible health risks for consumers.

## 1. Introduction

During the 19th and 20th centuries, humanity was driven by modern technical development and rapid urbanization. Since water is essential for life and its presence was regularly needed also for industrial development, most urban centres evolved next to the main water sources. This led to water pollution and caused many disease outbreaks [1]. Increasing knowledge in the fields of medicine, microbiology and chemistry established the connection between the presence of pathogens in water with diseases and implemented water cleaning procedures and the necessity for water quality monitoring [2]. These procedures prevented many deaths and contributed to a prolonged life span. Nowadays human population faces different threats. The world is quickly changing due to global warming [3], increasing globalisation, industrialisation, and growing population. Care for enough safe drinking water remains one of humanity’s most important global goals [4].

The current legislation includes the monitoring of many microbes, particularly the causative agents of gastrointestinal illnesses, however, listed global changes also promote the growth of other microorganisms, recognized as emerging pathogens, but not yet included in drinking water regulations [5,6]. Among them, the members of the fungal kingdom stepped into the forefront [7].

The presence of fungi in water has been known for a long time. Usually, the fungi have been connected to changes in taste, color, and odor of water [4,8,9], but lately, they have become increasingly recognized as emerging opportunistic pathogens, raising health concerns [4]. Mixed fungal bacterial biofilms often cause problems in industries, depending on the water sources used [8]. Nevertheless, there is still no uniform legislation on the building materials used in safe drinking water production. These choices depend on the country’s authorities and industrial financial budget [10,11]. Another aspect is the sustainable use of water and energy, that in combination with bio-based or recycled materials, often promotes the growth of sturdy fungal biofilms [12,13]. The World Health Organisation (WHO) reported and associated with contamination of wet materials species *Aspergillus flavus, A. versicolor, Cladosporium cladosporioides, C. herbarum, C. sphaerospermum, Mucor circinelloides* and *Rhizopus oryzae* [14]. Moreover, species of the genera *Acremonium, Alternaria, Candida, Debaryomyces, Fusarium, Kluyveromyces, Meyerozyma*, *Rhodotorula, Penicillium, Stachybotrys* and *Trichoderma* were reported as biodegraders of materials used for water transport [15,16]. The most commonly isolated fungi on the damp walls covered with plaster, wallpaper, and gypsum were *Acremonium* spp., *Penicillium chrysogenum*, *Stachybotrys* spp., *Ulocladium* spp., and *Phoma* spp. On the contrary, concrete coatings were more likely colonized by the fungi *Sporothrix* spp., *Chaetomium* spp., and *Penicillium* spp. In addition, *Aspergillus fumigatus, A. melleus, A. niger, A. ochraceus, Chaetomium* spp., *Mucor racemosus*, and *M. spinosus* [15] are often present on the concrete, final layers and adhesives (cork, linoleum and glue). The ubiquitary present fungal genus, regardless of the type of damaged material, was *Penicillium* [15]. Plastic materials, silicone, and rubber are colonized by *Candida* spp., *Cystobasidium sloofiae, Magnusiomyces capitatus, Meyerozyma guilliermondii, Naganishia* spp., *Rhodotorula mucilaginosa*, and melanized black yeasts *Phialophora* spp. and *Rhinocladiella similis* [4]. Biodegradation and deterioration of these materials are due to fungal production of organic acids [17,18], precipitation of minerals, leaching, and after extended periods of fungal growth also mechanical degradation (e.g., scaling, peeling, cracking) of the material [19]. These changes do not only cause water leakage and expenses due to clogging; they also negatively affect chlorination, taste, color, and odor of water [8]. 

The aim of the present study was to determine the presence, abundance, and diversity of fungi in chlorinated water entering 1.5 years old concrete water storage tanks already showing degradation, seen as local scaling and discoloration. We isolated and identified fungi actively involved in the colonization and degradation of ceilings in these tanks. Additionally, we followed the possible transmission of these fungi in the water distribution system, to the tap water end, in order to assess their potential effect on consumers’ health. 

## 2. Materials and Methods

### 2.1. Sampling of Drinking Water and Materials in Contact with Drinking Water, and Cultivation of Fungal Strains

**Figure 1:**
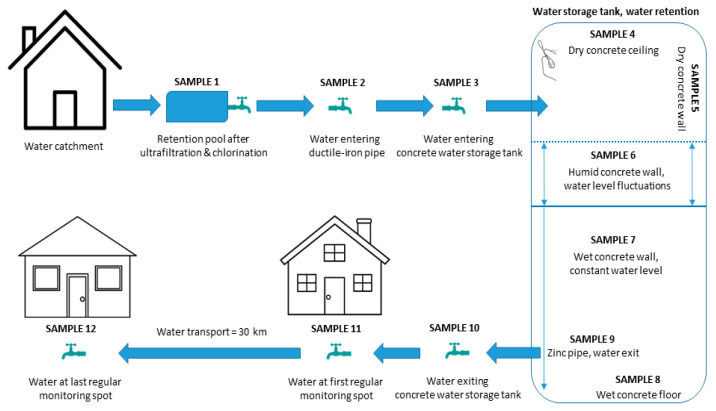
Sampling spots throughout water distribution system and inside concrete water storage tanks. Water samples are indicated as Sample-1, to -3, and Sample-10 to -12. Thick blue arrows indicate water flow. Samples inside the two concrete tanks that were taken by swabbing the surface are indicated as Sample-4 to -9. Thin blue arrows indicate water level as fluctuating or permanent.

Samples of 1.5 L of water were taken from 8 sampling points (Figure 1) in sterile containers as described by the standard SIST ISO 5667-5:2007. Samples were transported to the laboratory and processed immediately. Physico-chemical parameters of water samples were measured as required in Drinking Water Directive (98/83/EC) [5] at Komunala Novo mesto d.o.o., Slovenia, by trained personnel. Parameters included temperatures of water and air, concentrations of disinfectant, electric conductivity, pH, color, smell, taste, turbidity, and visible presence of sediment. 

For analysis of fungal presence, aliquots of 100 mL were filtered through 0.45 mm membrane filters (Merck, Millipore, Darmstadt, Germany). Filters were placed on solid media Dichloran Rose Bengal Agar (DRBC; Oxoid Ltd., Oslo, Norway) and Malt Extract Agar with the addition of chloramphenicol (MEA+Ch) to prevent bacterial growth. After visible growth of fungi, colonies on 10 aliquots of 100 mL were counted and colony-forming units per liter (CFU/L) were calculated. 

Samples from concrete and metal materials in contact with drinking water were taken from water storage tanks in Lutrško selo and Gorenja vas (both Novo mesto, Slovenia) by rubbing 1 cm^2^ of area with sterile cotton swabs (Figure 1), soaked in sterile saline solution. Swab samples were inoculated in duplicates by rubbing the whole area of the DRBC and MEA+Ch media.

All plates were incubated at 25 °C and 37 °C for 5 to 7 days. After visible growth of fungi, colonies were counted and colony-forming units (CFU/L and CFU/cm^2^) were calculated. All morphologically different strains were transferred to fresh Malt Extract Agar (MEA) plates and later permanently stored in the Ex Culture Collection of the Infrastructural Centre Mycosmo, MRIC UL, Slovenia (http://www.ex-genebank.com/), at the Department of Biology, Biotechnical Faculty, University of Ljubljana.

### 2.2. Extraction of Genomic DNA and Identification of Fungi

All cultures were grown on MEA prior to the extraction of the genomic DNA. DNA of filamentous fungi was extracted from 5 days old cultures by mechanical lysis of mycelia as recommended by Van den Ende and de Hoog [20]. DNA from 3 days old yeast cultures was extracted with PrepMan Ultra reagent (Applied Biosystems, Foster City, USA) according to the manufacturer’s instructions. Obtained DNA samples were stored at −20°C till further use. 

Depending on their morphological characterization, complete identification of filamentous fungi based either on the rDNA nucleotide sequence analyses of the whole internal transcribed spacer region (ITS: ITS1, 5.8S rDNA and ITS 2), partial beta-tubulin gene exons and introns (*benA*), or the partial actin gene (*act*). Yeasts were identified by their large subunit ribosomal DNA (LSU) sequence (partial 28S rDNA, D1/D2 domains). Amplification and sequencing were carried out with primer pairs ITS5 and ITS4 [21], Bt2a and Bt2b [22], ACT-512F and ACT-783R [23], or NL1 and NL4 [24]. Sequences were obtained at Microsynth AG, Austria and assembled by FinchTV 1.4 (Geospiza, PerkinElmer, Inc., Seattle, USA). The alignments were adjusted using Molecular Evolutionary Genetics Analysis (MEGA) software version 7.0 [25] and identified with the BLAST algorithm on the NCBI web page [26]. Final identification was supported by taxonomically important databases such as Westerdijk Fungal Biodiversity Institute (Utrecht, the Netherlands) and Index Fungorum (www.indexfungorum.org). The sequences of representative strains used in the study were deposited in the GenBank database (NCBI). 

### 2.3. Fungal Growth under Oligotrophic Conditions and Their Biodegradation Potential 

Based on their taxonomy and location of isolation, 52 different strains were selected for further analysis. They were tested at different environmental conditions, to determine whether water and materials serve only as vectors for transmission or promoted fungal growth. Fungal adaptation to oligotrophic conditions was tested on 100-times diluted Yeast Nitrogen Base (YNB) solid medium and 2% water agar medium (WA). 

In order to check their biodegradation and biodeterioration potential calcium carbonate (CaCO_3_) agar plates [27] were used. Additionally, changes in pH were measured after incubation in liquid medium with neutral pH [28] using Seven Compact S210 (Mettler Toledo, Switzerland). Growth of fungi in real-life conditions was tested on a commercial product used for concrete coatings (Xypex Concentrate, XYPEX CE s.r.o.), consisting of portland cement (30–60%), alkaline earth compounds (10–30%), silica sand (30–60%) and calcium aluminates (<10%). Xypex plates were prepared according to the manufacturer’s instructions and wetted with 5 mL of sterile water prior to use. 

Fungal cell suspensions were prepared from 3–5 days old cultures in a sterile saline solution and adjusted to ~1.0 × 10^8^ CFU/mL. Ten microliters of the suspensions were inoculated on all media and incubated at 15 °C for 1 month. One series of inoculated YNB medium was used for testing the ability of strains to grow at human body temperature (37 °C, 1 month). Xypex Concentrate plates were incubated at 15 °C up to 3 months. After incubation, growth of fungi was checked and described as very good (+++) when mycelium covered > 70% of the plate, good (++; mycelium covered 41–70% of the plate), weak (+; mycelium covered 20–40% of the plate), poor (-/+; mycelium covered < 20% of the plate) or none (-; no growth was observed).

## 3. Results

### 3.1. Fungi Are Present at Every Stage of Drinking Water Preparation

Drinking water was sampled at eight different locations: from a pool after ultrafiltration (1), from a pipe at the beginning of transport toward water storage tanks (2), at the entrance of water storage tanks (3a and 3b), at the exit of water storage tanks (4a and 4b), at the first sampling point for water monitoring (5), and at the last sampling point for regular water monitoring (6) (Figure 1). All tested physico-chemical parameters of water samples were in normal range set by Drinking Water Directive (98/83/EC), e.g., water hardness = 15.2 N (medium hard water), electrical conductivity at 20 °C = 423–430 µS/cm, total organic carbon (TOC< 0.3 mg/L), Ammonium < 0.01 mg/L, Nitrite < 0.001 mg/L, Nitrate = 3.92–4.53 mg/L, Manganese <0.1–0.14 µg/L, Iron < 40 µg/L, Aluminum = 5.5–6.1 µg/L, Arsenic = 0.49–0.54 µg/L, Copper = 0.0035–0.0078 mg/L, Cadmium <0.02 µg/L, Chromium = 0.54–0.57 µg/L, Nickel < 0.1 µg/L, and Lead = 0.27–0.34 µg/L. The pH, measured at temperatures 20–21°C was slightly alkaline, between 7.3 and 7.5. Additionally, chlorination performed with gaseous chlorine (Cl_2_) did not show any discrepancies, with amounts of residual chlorine within the recommended range at all eight sampling points (between 0.15 and 0.30 mg/L). 

**Figure 2:**
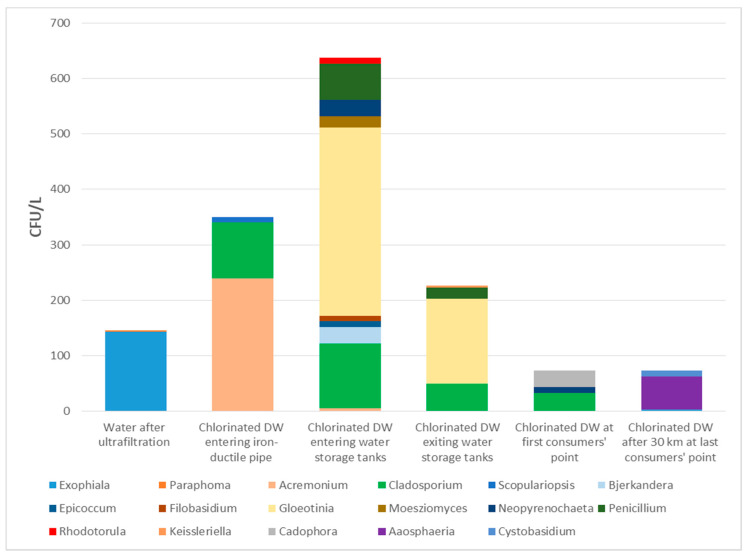
Presence and diversity of fungal genera throughout water distribution network. Drinking water (DW) was sampled at eight distant locations. Both, the diversity and the numbers (CFU/L) were highest at the point of entrance into the concrete water storage tanks and the lowest at consumers’ endpoints (taps), indicating sensitivity to chlorine under prolonged exposure time. The genus *Cladosporium* was the most frequently isolated (four out of eight sampling spots).

Fungi were isolated from all water samples (Table 1). The most frequently isolated genus was *Cladosporium*, detected in four out of eight sampling locations (50%). One-third of the samples were positive for filamentous fungi of the genera *Acremonium, Exophiala, Gloeotinia, Neopyrenochaeta*, and *Penicillium*, while yeasts were detected only sporadically. Some fungi were not detected throughout the water preparation process. For instance, water from the retention pool yielded after ultrafiltration 146 CFU/L, 143 of them were identified as *Exophiala cancerae*. Nevertheless, this species was completely absent at later stages that followed water chlorination. The number of fungi in chlorinated drinking water was the highest before entering the main pipe system toward water storage tanks and at the immediate entrance to water storage tanks (both uncountable; > 330 CFU/L). The most common fungus in water before entering the pipe system was *Acremonium sclerotigenum* (160 CFU/L), while *Gloeotinia* sp. dominated in samples just before entering the water storage tanks (> 330 CFU/L) and at the exit point of the tanks (153 out of 226 CFU/L). Numbers lowered with the distance and time of water transportation. For instance, species of *Cladosporium* and *Penicillium* were present in water entering and exiting the storage tanks, while only *Cladosporium* was detected also at the first consumers’ tap sampling point. The lowest number of fungi was detected in the samples at the first and the last regular monitoring spot at consumers’ taps (both 73 CFU/L). *Cadophora malorum* was with 30 CFU/L the most common fungus at the first consumers’ tap collection point, and *Aaosphaeria arxii* (60 CFU/L) was the most common species after 30 km of water flow at the last consumers’ tap collection point (Table 1 and Figure 2).

### 3.2. Number and Diversity of Fungi Inside Two Water Storage Tanks

**Figure 3:**
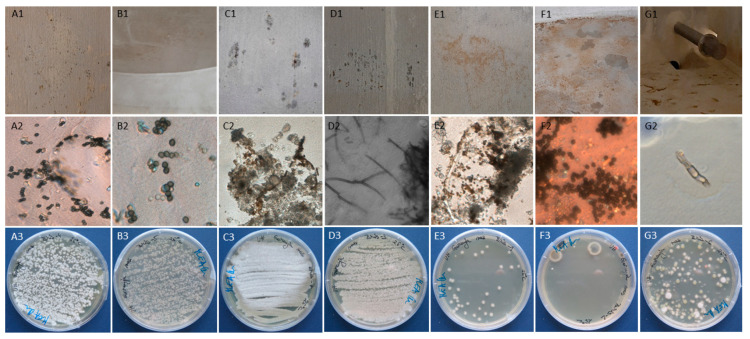
Presence of fungi inside concrete water storage tanks. Fungal growth with visible mycelia was observed as black and ochre colonies on concrete ceilings at the entrance (**A1**) and inside the cell (**B1**), on a dry wall (**C1**), humid wall (**D1**), and wet wall (**E1**). Samples were taken also from the bottom of the tank (**F1**) with visible peeling and change of the colour and from the metal pipe for water outflow (**G1**). (**A2**–**G2**): Microscopic observation of adhesive tape samples revealed diverse mycobiota producing brown conidia and brown to hyaline hyphae at all sampling sites. (**A3**–**G3**): Fungal colonies on MEA medium grown from swab samples taken from 1 cm^2^ surface.

Both concrete water storage tanks were filled with drinking water of the same origin, following the same preparation procedure. Although only 1.5 years old, both showed local degradations like cracks and peeling. Additionally, walls below the constant water level changed the color from grey to ochre. At some points growth of visible fungal mycelia was observed (Figure 3). All samples from the surfaces of water storage tanks were positive for fungi and together yielded 23 isolates. Their numbers were the highest on a dry ceiling (Samples-4a and 4b), locally covered with condensate of water drops. There several species (*Akanthomyces muscarius, Aspergillus protuberus, Cladosporium sphaerospermum, Musicillium elettariae* and *Sarocladium kiliense*) co-occurred in more than 330 CFU/cm^2^ (Table 1). An uncountable number of fungal colonies per cm^2^ was observed also on humid walls of water storage tanks, where water level varies depending on the production/consumption ratio of water. Contrary to the dry ceiling with high numbers and diversity of fungi, samples taken from humid walls yielded monoculture of *Furcasterigmium furcatum*. This species was present also on dry (Samples-5a and 5b) and completely wet walls (Samples-7a and 7b) and likely grew also on concrete under constant water level. *Furcasterigmium* often co-existed with *Sarocladium kiliense,* also present on wet walls under constant water level, and on the surface of the metal pipe for water exit inside the tanks. The least contaminated part of the concrete water storage tanks was the bottom, with only few colonies of conidiogenic fungi per cm^2^. The water pressure on the bottom is higher and oxygen level is lower in comparison to upper sampled locations (Table 1). 

### 3.3. Oligotrophic and Aerophilic Conditions Promote Fungal Growth

All tested strains incubated for a month at 15 °C grew well on both oligotrophic media, YNB and WA, mimicking low-nutrient conditions inside water-networks and water storage tanks. However, their growth was slower on Xypex Concentrate plates, incubated at 15 °C for 3 months. Only 40.4% of the strains grew weakly (+), 13.5% grew poorly (-/+), while 46.1% of the strains did not grow at these conditions (Table 2). 

The growth of fungi at different aerobic conditions was determined by the immersion of the mycelium in liquid pH medium in glass tubes without shaking. Fifty percent of tested strains formed mycelium between the top to the middle of glass tubes, 32.7% strains grew throughout the whole medium column, and 17.3% grew from the middle to the bottom of the testing tubes (Table 2). These results are in accordance with the numbers and diversity of fungi isolated from water storage tanks. Most of these fungi grew better on well-aerated ceilings, upper and middle walls, while the lowest number was detected on the bottom of the retention tanks. 

### 3.4. Two-Thirds of Water-Related Strains Produce Acidic Metabolites 

Production of acidic or alkaline fungal metabolites can have a long-term effect on the biodeterioration of materials. Possible biodeterioration ability of isolated strains was tested in liquid medium with neutral pH and on solid medium with added calcium carbonate (CaCO_3_) and glucose. After incubation in the pH medium, 32.7% of strains were classified as producers of alkaline metabolites, since pH rose in a range of 0.01 to 0.5 units. On the other hand, 67.3% of tested strains acidified the medium, most of them (55.8%) lowered pH in a range between 0.01 and 0.5 units and 3.8% lowered pH in a range of 0.5 to 1.5 units (Table 2). *Penicillium* strains (*P. bialowiezense, P. brevicompactum,* and *P. glabrum;* representing 7.7%) were the best producers of acidic metabolites, lowering pH between 1.5 to 3.0 units. All these penicillia also actively dissolved calcium carbonate on solid plates, with a clearing zone appearing after incubation. Besides *Penicillium*, only four other strains (*A. muscarius, Gloeotinia* sp., *N. fragariae*, and *T. versicolor*) clearly dissolved calcium carbonate, shown as the formation of clearing zones (Table 2). Additionally, *Gloeotinia* sp. was the only strain that changed during growth on calcium carbonate the color of the mycelium from white-yellowish to ochre.

### 3.5. Water-Borne Fungi and Possible Effect on Human Health 

The ability of fungal strains to grow at 37 °C is the most important virulence factor for possible colonization of the human body [8]. Thermotolerant potential of representative strains was thus tested on solid plates with 100-times diluted YNB, incubated at 37°C for 1 month. After incubation, 5.8% of the strains expressed very good (+++) growth, 9.6% good growth (++), and 3.8% weak growth (+). Poor growth was observed for 11.5% of the strains, while 69.3% of the strains did not grow at this temperature at all (Table 2). Best growth was observed for *A. sclerotigenum* and *Moesziomyces aphidis*, followed by *F. furcatum* and *S. kiliense*. 

## 4. Discussion

Preparation of drinking water in EU countries depends on the availability of the main water source and is strictly monitored as requested by Drinking Water Directive (98/83/EC) [5]. Predominant geological structures within the territory of Slovenia are sedimentary rocks and sediment deposits, which are ideal for the development of aquifers [29]. Due to the favorable geological location and relatively easy access, groundwater is the main raw water source in Slovenia. In total, 85% of the country’s population drinking water is supplied with groundwater, and only 3% of the population uses surface or rainwater. The rest use water from their own wells [30,31]. The geological specialty of Slovenia is the karst system, covering > 44% of the territory [29]. Underground karstic aquifers occur due to their limestone structure and thin layers that are very sensitive to anthropogenic pollution that may affect water quality [30]. To meet the microbiological criteria set in Drinking Water Directive (98/83/EC), out of 866 active Slovenian water supply areas (WS), 31% do not need disinfection for water preparation, 9% use disinfection procedures occasionally, and 60% have permanent disinfection [31]. Similar to other countries, the most common chemical disinfection process in Slovenia is chlorination with sodium hypochlorite (NaOCl), chlorine gas (Cl_2_), and chlorine dioxide (ClO_2_), being used in 40%, 14.1% and 2.2% of water supplies, respectively. During the last years also use of non-chemical UV disinfection increased [31].

Drinking water samples analyzed in the study were obtained from the karst area where the raw water source originates as groundwater below thin layers of limestone. The water supply company has installed a modern system of pre-treatment with combined ultrafiltration and disinfection with chlorine gas. Additionally, new ground concrete reservoirs for water storage and distribution have been built. However, after 1.5 years of regular use, cement coatings inside concrete water storage tanks showed changes in color from white to ochre, with sporadically observed local peelings. Additionally, also visible is contamination resembling mold colonies appeared on the upper ceilings and walls. Nevertheless, all microbiological and chemical parameters measured for the regular drinking water monitoring were within the normal range as set in Drinking Water Directive (98/83/EC) [5]. The reasons for following the occurrence and diversity of fungi were (i) possible impact on material degradation and (ii) possible impact on consumers’ health. 

In order to find out whether fungi caused discoloration, peeling and visible contamination of upper ceilings and walls, six different surface spots inside the two water retention tanks filled with drinking water of the same origin were sampled. Together 12 sampling spots of the tanks’ surface were sampled. Additionally, samples of drinking water were taken from eight points throughout the water network system to follow related fungal contamination. All samples of drinking water and storage tanks were positive for fungi. Since there is currently no uniform parametric value for fungi in drinking water, we compared our results with the Swedish legislation [32], which limits the presence of fungi to 100 CFU per 100 mL (equivalent to 1000 CFU/L). None of the samples exceeded this number. However, the highest number estimated as ~637 CFU/L and diversity of fungi were determined in water at the entrance of water storage tanks, after being transported from the Water Company through the old system constructed of iron-based pipes. The reason for the high number of fungi could be their presence in well-established biofilms inside the old pipes [13,33]. Their number lowered already at the outflow of the water storage tanks and was the lowest at both consumers’ endpoints. The lowered counts are likely due to chlorination since residual chlorine was present throughout the entire system [34,35,36]. Although mycobiota differed between the sampling locations, isolated species of the genera *Acremonium, Cadophora, Cladosporium, Epicoccum, Penicillium* and *Rhodotorula* represented the “core-species”, as described previously in other EU countries, confirming drinking water as an important vector for their transmission [4,6,8]. These fungal genera are also common in surface water or water from karst systems with thin layers of sediments [4,37]. Surprisingly, we did not isolate *Aspergillus, Aureobasidium*, or *Candida* species, which otherwise represent dominant groups of fungi in groundwater-derived drinking water in Slovenia [38]. The reason could be their susceptibility to chlorination [34,35,36,39,40] or the mixed source of water as is common in karstic systems. We additionally successfully isolated yeasts belonging to the genera *Cystobasidium, Filobasidium, Moesziomyces* and *Rhodotorula*. The ratio between isolated yeasts vs. filamentous fungi was 1:29 in favor of the filamentous fungi, in accordance with studies of biofilms in drinking water distribution systems [33,41]. Despite relatively high numbers of different fungi in the entering water, only *Acremonium, Cadophora, Cladosporium,* and *Paraphoma* were isolated from both waters, and the surfaces of water storage tanks. Since sampling was performed only once as a case study, the reasons for a limited number of species found on both surfaces and drinking water may be the fluctuation in mycobiota in the water at the time of the sampling [42]. 

Main pipes, large retention centres and modern water storage tanks are made of rough concrete covered with coatings to prevent leaching and corrosion of metals used in concrete [19]. After the relative humidity of the material in constant contact with water raises above 80% it represents an ideal scaffold for secondary colonizers like *Aspergillus flavus, A. versicolor, Cladosporium cladosporioides, C. herbarum, C. sphaerospermum, Mucor circinelloides* and *Rhizopus oryzae* [14]. With relative humidity above 90%, wet material promotes the growth of a variety of water-related hydrophilic species [14]. Another important factor affecting the growth of fungi on wet materials is the presence of oxygen. Most fungi are aerobes, some are microaerophilic, but both groups need oxygen for the synthesis of cell membrane constituents, like ergosterol and unsaturated fatty acids. Additionally, oxygen is needed in the respiratory chain as the terminal electron acceptor [43]. Considering both, the high relative humidity and fungal need for oxygen, it can be expected that fungi would more likely colonize ceilings and upper humid walls of concrete water storage tanks. Wet concrete and finishing materials thus often harbor the genera *Acremonium, Cladosporium, Aspergillus, Bjerkandera, Sporothrix, Chaetomium, Mucor, Stachybotrys, Ulocladium, Phoma* and *Penicillium.* Yeasts on concrete are less diverse and belong mainly to the genera *Candida, Debaryomyces, Kluyveromyces, Meyerozyma* and *Rhodotorula* [15,16]. Our results, both in diversity and numbers of fungi, are in accordance with previous studies, with *Aspegillus, Cladosporium*, and *Sarocladium, Furcasterigmium* and *Musicillium* (all previously described as *Acremonium*) being the most common. Their numbers were highest on well-aerated dry ceilings and upper walls with locally visible condensate and lower on humid walls and walls constantly under water level (Table 1). The need for oxygen was confirmed with the test in liquid medium without shaking. Species of *Akanthomyces, Aspergillus* and *Cladosporium,* found on upper ceilings and walls of storage tanks grew only on the top of the medium. On the contrary, *Musicillium, Sarocladium* and *Furcasterigmium*, isolated from dry and humid walls, as well as from the walls under constant water level, grew throughout the whole medium column, from totally aerobic to microaerophilic conditions (Table 2). 

After a prolonged time of use, materials react with chemical components and disinfectants in water and become more susceptible to biofilm formation and biodegradation [1,8]. The presence and growth of fungi in water and on concrete may affect the material via the production of organic acids [17,18] causing local scaling. The process often involves *Aspergillus*, *Penicillium*, *Fusarium*, *Paecilomyces, Talaromyces, Stachybotrys,* and zygomycetous fungi [15,44]. The opposite process, known as “self-healing concrete”, was confirmed for *Trichoderma reesei* and *Candida tropicalis* and starts with the dissolution of Ca(OH)_2_, leading to the precipitation of calcite on the surface of fungal cells [44,45]. Production of acidic or alkaline products of isolated fungal strains was tested in liquid medium with neutral pH. Two-thirds of tested strains produced acidic metabolites, with the genus *Penicillium* being able to lower pH up to three units (Table 2). These strains also displayed the largest clearing zones on the solid medium with added calcium carbonate (CaCO_3_) and glucose, while among other producers of acidic compounds only four strains additionally dissolved CaCO_3_ (Table 2), indicating additional mechanisms, besides acid production, affecting the solubility of calcium carbonate. Among isolated strains, only *Gloeotinia* sp. produced acidic metabolites, dissolved calcium carbonate, and changed the color of mycelium from white-yellowish to ochre. 

Following chemical dissolution, hypothetically, in-depth growth of hyphae causes mechanical degradation of the material [19], as described for water-related genera *Alternaria, Aspergillus*, *Cephalosporium, Cladosporium, Mucor, Penicillium, Rhizopus,* and *Trichoderma* [44,46]. When using Xypex Concentrate as the only source of nutrients, only 40.4% of the strains showed weak growth, seen as thin mycelium on the surface of the plates. Since the test was carried out only for 3 months, with single strains under highly oligotrophic conditions, these fungi nevertheless showed the potential for biomechanical degradation, particularly over a prolonged time, in an environment contaminated with a mixed biofilm and in the presence of additional nutrients carried by water. 

Although the presence of fungi can induce, as side effects, chemical dissolution and mechanical degradation of material, observed as changes of odor and taste of drinking water, these changes alone do not pose danger to human health [8,47]. One of the main worries is the potentially elevated number of opportunistic and/or mycotoxigenic fungi. Fungal ability to colonize the human body does not depend on fungal physiology only, but greatly also on the immune defense of the host [48] and the ability of strains to grow at 37 °C. In the case of tested strains from drinking water and storage tanks, 29.7% grew at 37 °C, and only 7.7% were classified under Biosafety Level 2 (e.g., *Sarocladium kiliense* and *Filobasidium uniguttulatum*). However, none of them was detected at consumers’ taps, indicating a low risk for fungal infections through the sampled water.

Mycotoxins are well described in agriculture and food production, but there are still few data about mycotoxins in water. One of the issues may be their detection and quantification, because only a few are hydrophilic (e.g., fumonisins), while most known toxins are hydrophobic [49]. Chlorination inactivates some mycotoxins (e.g., aflatoxin B1), and recent studies reported low levels of mycotoxins in European chlorinated water, but the caution must be maintained in cases of low- or unchlorinated water, particularly when drawn from surface water sources or water sources of mixed origin [6,50,51].

## 5. Conclusions

Building materials in contact with drinking water must be, on one hand, safe for use, and on the other hand, able to sustain quick degradation processes. Yet, many chemical and biological agents present in nature can fasten their degradation. We investigated the presence of fungi, known degraders of recalcitrant materials, inside affected modern concrete water storage tanks. The tanks were filled with chlorinated drinking water originating from a karstic water source. The majority of isolated fungi were able to grow under water level, produced acids and dissolved carbonate media, thus fungi should be taken into consideration also as causative agents of visible coloration and peeling of retention tank surfaces in contact with drinking water. Fungal abundance, followed from storage tanks throughout the distribution system, gradually diminished and was the lowest at consumers’ taps. No known opportunistic pathogens were detected, yet they need to be taken into consideration in cases with visible overgrowth of mycelia inside retention tanks. 

## Figures and Tables

**Table 1:** Number and diversity of fungi isolated from drinking water through distribution network and from concrete water storage tanks.

Sample Number	Description	Total Number of Fungal Colonies	Number of Colonies per Species	Genus	Species	EXF- No *
Sample -1	Water from retention pool after ultrafiltration	146 CFU/L	143 ^+^CFU/L	Exophiala	cancerae	14903
			3 CFU/L	Paraphoma	radicina	14904
Sample-2	Chlorinated drinking water entering iron-ductile pipe leading toward water storage tanks	350 CFU/L	80 CFU/L	Acremonium	charticola	14755
			160 CFU/L	Acremonium	sclerotigenum	14749, 14750, 14751, 14791
			100 CFU/L	Cladosporium	sphaerospermum	14744
			10 CFU/L	Scopulariopsis	brumptii	14743
Sample-3a (water storage tank 1) and 3b (water storage tank 2)	Chlorinated drinking water entering water storage tanks	estimated as > 627 CFU/L	5 CFU/L	Acremonium	sclerotigenum	14916
			30 CFU/L	Bjerkandera	adusta	14740
			10 CFU/L	Cladosporium	allicinum	14572
			7 CFU/L	Cladosporium	cladosporioides	14917
			20 CFU/L	Cladosporium	halotolerans	14567
			70 CFU/L	Cladosporium	pseudocladosporioides	14566, 14575
			10 CFU/L	Cladosporium	sphaerospermum	14573
			10 CFU/L	Epicoccum	nigrum	14736
			10 CFU/L	Filobasidium	uniguttulatum	14741
			> 330 CFU/L	Gloeotinia	sp.	14568, 14738, 14739
			20 CFU/L	Moesziomyces	aphidis	14742
			30 CFU/L	Neopyrenochaeta	fragariae	14737, 14754
			35 CFU/L	Penicillium	brevicompactum	14918
			10 CFU/L	Penicillium	glabrum	14569, 14570
			20 CFU/L	Penicillium	verhagenii	14571
			10 CFU/L	Rhodotorula	mucilaginosa	14574
Sample-4a (water storage tank 1) and 4b (water storage tank 2)	Dry concrete ceiling inside water storage tanks	> 330 CFU/cm^2^	90 CFU/cm^2^	Akanthomyces	muscarius	14589
			> 330 CFU/cm^2^	Aspergillus	protuberus	14928, 14929
			> 330 CFU/cm^2^	Cladosporium	sphaerospermum	14927
			> 330 CFU/cm^2^	Musicillium	elettariae	14931
			> 330 CFU/cm^2^	Sarocladium	kiliense	14930
Sample-5a (water storage tank 1) and 5b (water storage tank 2)	Dry concrete wall inside water storage tanks	11 CFU/cm^2^	5 CFU/cm^2^	Furcasterigmium	furcatum	14586, 14587
			6 CFU/cm^2^	Sarocladium	kiliense	14585
Sample-6a (water storage tank 1) and 6b (water storage tank 2)	Humid concrete wall inside water storage tanks	> 330 CFU/cm^2^	> 330 CFU/cm^2^	Furcasterigmium	furcatum	14926
Sample-7a (water storage tank 1) and 7b (water storage tank 2)	Wet concrete wall inside water storage tanks	94 CFU/cm^2^	56 CFU/cm^2^	Furcasterigmium	furcatum	14583, 14584
			34 CFU/cm^2^	Musicillium	elettariae	14922
			4 CFU/cm^2^	Sarocladium	kiliense	14582
Sample-8a (water storage tank 1) and 8b (water storage tank 2)	Wet concrete bottom inside water storage tanks	9 CFU/cm^2^	4 CFU/cm^2^	Acremonium	camptosporum	14923
			3 CFU/cm^2^	Cladosporium	pseudocladosporioides	14924
			2 CFU/cm^2^	Trametes	versicolor	14925
Sample-9a (water storage tank 1) and 9b (water storage tank 2)	Metal pipe for water exit at the bottom of the water storage tanks	124 CFU/cm^2^	23 CFU/cm^2^	Cadophora	malorum	14934
			1 CFU/cm^2^	Emericellopsis	sp.	14936
			9 CFU/cm^2^	Paraphoma	radicina	14933
			58 CFU/cm^2^	Sarocladium	kiliense	14935
			33 CFU/cm^2^	Stereum	armeniacum	14932
Sample-10a (water storage tank 1) and 10b (water storage tank 2)	Chlorinated drinking water exiting water storage tanks	226 CFU/L	10 CFU/L	Cladosporium	allicinum	14578
			10 CFU/L	Cladosporium	halotolerans	14577
			30 CFU/L	Cladosporium	perangustum	14579
			153 CFU/L	Gloeotinia	sp.	14576, 14580, 14920
			3 CFU/L	Keissleriella	caudata	14921
			10 CFU/L	Penicillium	bialowiezense	14581
			10 CFU/L	Penicillium	brevicompactum	14919
Sample-11	Chlorinated drinking water at the first standard monitoring sampling point	73 CFU/L	30 CFU/L	Cadophora	malorum	14747
			13 CFU/L	Cladosporium	allicinum	14746
			20 CFU/L	Cladosporium	pseudocladosporioides	14745
			10 CFU/L	Neopyrenochaeta	acicola	14748
Sample-12	Chlorinated drinking water at the last standard monitoring sampling point (after 30 km of flow)	73 CFU/L	60 CFU/L	Aaosphaeria	arxii	14906, 14907
			10 CFU/L	Cystobasidium	lysinophilum	14905
			3 CFU/L	Exophiala	equina	14908

Legend: * EXF- No.: number of fungal strains deposited in the EX Culture Collection in of the Infrastructural Centre Mycosmo, at the Department of Biology, Biotechnical Faculty, University of Ljubljana. ^+^ CFU: colony-forming units; fungal particles, spores, and conidia germinating in a new colony.

**Table 2:** Representative fungal strains isolated from water storage tanks and drinking water, used to test the growth in vitro.

Genus	Species	EXF-No.	GenBank No.	Growth after 1 Month							Growth after 3 Months
				0.01 x YNB		2% WA	CaCO_3_ Medium		pH Medium		Xypex Medium
				15 °C	37 °C	15 °C	15 °C	Dissolution	pH Change	Position of Mycelium	15 °C
*Aaosphaeria*	*arxii*	14906	MT178773 (ITS)	+++	-	+++	+++	-	↓ 0.41	throughout	-
*Acremonium*	*camptosporum*	14923	MT178774 (ITS)	+++	-	++	+++	-	↓ 0.15	top to middle	-
*Acremonium*	*charticola*	14755	MT178775 (ITS)	++	-	+++	++	-	↓ 0.24	throughout	+
*Acremonium*	*sclerotigenum*	14916	MT178776 (ITS)	+++	+++	+++	+++	-	↓ 0.22	throughout	+
		14749	MT178777 (ITS)	+++	+++	+++	+++	-	↓ 0.25	throughout	-/+
*Akanthomyces*	*muscarius*	14589	MT178778 (ITS)	+++	+	+++	+++	+++	↓ 0.40	top to middle	-/+
*Aspergillus*	*protuberus*	14928	MT178779 (ITS)	+++	-	+++	+++	-	↑ 0.36	top to middle	-
*Bjerkandera*	*adusta*	14740	MT178780 (ITS)	+++	-/+	+++	++	-	↓ 0.15	bottom to middle	+
*Cadophora*	*malorum*	14747	MT178781 (ITS)	+++	-/+	+++	+++	-	↓ 0.29	top to middle	-
		14934	MT178782 (ITS)	+++	-	+++	+++	-	↓ 0.33	top to middle	+
*Cladosporium*	*allicinum*	14578	MT239332 (*act*)	+++	-	+++	+++	-	↓ 0.05	top to middle	+
		14746	MT239333 (*act*)	+++	-	+++	+	-	↓ 0.12	bottom to middle	+
		14572	MT239331 (*act*)	+++	-	+++	+++	-	↑ 0.04	top	+
*Cladosporium*	*cladosporioides*	14917	MT239339 (*act*)	+++	-	+++	+++	-	↓ 0.07	top to middle	-
*Cladosporium*	*halotolerans*	14567	MT239334 (*act*)	+++	-	+++	+++	-	↑ 0.12	throughout	+
		14577	MT239335 (*act*)	+++	-	+++	+++	-	↑ 0.07	throughout	+
*Cladosporium*	*perangustum*	14579	MT239330 (*act*)	+++	-	+++	+++	-	↓ 0.34	top to middle	+
*Cladosporium*	*pseudocladosporioides*	14566	MT239336 (*act*)	+++	-	+++	+++	-	↑ 0.41	top	+
		14745	MT239337 (*act*)	+++	-	+++	+++	-	↓ 0.04	top	+
		14924	MT239338 (*act*)	+++	-	+++	+++	-	↑ 0.28	top	+
*Cladosporium*	*sphaerospermum*	14573	MT239340 (*act*)	+++	-	+++	+++	-	↓ 0.21	top to middle	+
		14744	MT239341 (*act*)	+++	-	+++	+++	-	↓ 0.04	top to middle	-
		14927	MT239342 (*act*)	+++	-	+++	+++	-	↓ 0.07	top to middle	+
*Cystobasidium*	*lysinophilum*	14905	MT154654 (LSU)	+++	-/+	++	+	-	↑ 0.11	bottom	-
*Emericellopsis*	sp.	14936	MT178783 (ITS)	+++	-	+++	++	-	↓ 0.19	top to middle	+
*Epicoccum*	*nigrum*	14736	MT178784 (ITS)	+++	-	+++	+++	-	↑ 0.05	bottom	+
*Exophiala*	*cancerae*	14903	MT178785 (ITS)	+++	-	+++	+++	-	↑ 0.05	throughout	+
*Exophiala*	*equina*	14908	MT178786 (ITS)	+++	-	+++	+++	-	↑ 0.07	throughout	+
*Filobasidium*	*uniguttulatum*	14741	MT154655 (LSU)	+++	+	++	++	-	↓ 0.03	bottom	-/+
*Furcasterigmium*	*furcatum*	14583	MT178787 (ITS)	+++	++	+++	+++	-	↓ 0.14	throughout	-
		14926	MT178788 (ITS)	+++	++	+++	+++	-	↓ 0.11	throughout	-
*Gloeotinia*	sp.	14920	MT178789 (ITS)	+++	-	+++	+++	+	↓ 0.50	throughout	-
*Keissleriella*	*caudata*	14921	MT178792 (ITS)	+++	-	+++	+++	-	↑ 0.12	top to middle	-
*Moesziomyces*	*aphidis*	14742	MT154656 (LSU)	++	+++	++	+	-	↓ 0.13	bottom	-
*Musicillium*	*elettariae*	14922	MT178793 (ITS)	+++	-/+	+++	+++	-	↓ 0.21	throughout	-/+
		14931	MT178794 (ITS)	+++	-/+	+++	+++	-	↓ 0.11	throughout	-/+
*Neopyrenochaeta*	*acicola*	14748	MT178795 (ITS)	+++	-	+++	+++	-	↑ 0.36	bottom to middle	-
*Neopyrenochaeta*	*fragariae*	14737	MT178797 (ITS)	++	-	+	+++	+++	↓ 0.44	top	-
		14754	MT178796 (ITS)	+++	-	++	+++	-	↑ 0.18	bottom to middle	-
*Paraphoma*	*radicina*	14933	MT178799 (ITS)	+++	-	+++	+++	-	↓ 0.03	top	-
		14904	MT178798 (ITS)	+++	-	++	+++	-	↓ 0.17	top	-
*Penicillium*	*bialowiezense*	14581	MT162690 (*benA*)	+++	-	+++	+++	+++	↓ 2.71	top to middle	-
*Penicillium*	*brevicompactum*	14918	MT162691 (*benA*)	+++	-	+++	+++	+++	↓ 2.24	top	-/+
		14919	MT162692 (*benA*)	+++	-	+++	+++	+++	↓ 1.66	top	-
*Penicillium*	*glabrum*	14569	MT162693 (*benA*)	+++	-	+++	+++	+++	↓ 2.18	top to middle	-
*Penicillium*	*verhagenii*	14571	MT162694 (*benA*)	+++	-	+++	++	-	↓ 0.33	top to middle	+
*Sarocladium*	*kiliense*	14582	MT178800 (ITS)	+++	++	+++	+++	-	↑ 0.02	throughout	-
		14930	MT178801 (ITS)	+++	++	+++	+++	-	↑ 0.10	throughout	-
		14935	MT178802 (ITS)	+++	++	+++	+++	-	↑ 0.05	throughout	-
*Scopulariopsis*	*brumptii*	14743	MT178803 (ITS)	+++	-	+++	++	-	↑ 0.14	throughout	+
*Stereum*	*armeniacum*	14932	MT178804 (ITS)	+++	-/+	++	-	-	↓ 0.79	top	-/+
*Trametes*	*versicolor*	14925	MT178805 (ITS)	+++	-	+++	+++	++	↓ 1.34	bottom to middle	-

Legend: Growth of fungi is described as very good (+++; mycelium covered > 70% of the plate), good (++; mycelium covered 41–70% of the plate), weak (+; mycelium covered 20–40% of the plate), poor (-/+; mycelium covered < 20% of the plate) or none (-; no growth was observed). Changes of pH from neutral into acidic zone are indicated with ↓, and into alkali zone with ↑.

## Data Availability

Not Applicable.
